# Real‐world data on treatment concepts in classical Hodgkin lymphoma in Sweden 2000–2014, focusing on patients aged >60 years

**DOI:** 10.1002/jha2.202

**Published:** 2021-05-06

**Authors:** Björn Engelbrekt Wahlin, Ninja Övergaard, Stefan Peterson, Evangelos Digkas, Ingrid Glimelius, Ingemar Lagerlöf, Ann‐Sofie Johansson, Marzia Palma, Lotta Hansson, Johan Linderoth, Christina Goldkuhl, Daniel Molin

**Affiliations:** ^1^ Division of Haematology, Department of Medicine, Huddinge Karolinska Institutet Stockholm Sweden; ^2^ Haematology unit Karolinska University Hospital Stockholm Sweden; ^3^ Department of Immunology, Genetics and Pathology Uppsala University Uppsala Sweden; ^4^ Regional Cancer Center South Lund Sweden; ^5^ Department of Oncology Mälarsjukhuset Eskilstuna Sweden; ^6^ Department of Haematology University Hospital of Linköping Linköping Sweden; ^7^ Department of Oncology Umeå University Umeå Sweden; ^8^ Department of Oncology Lund University Lund Sweden; ^9^ Department of Oncology Sahlgrenska University Hospital Gothenburg Sweden

**Keywords:** classical Hodgkin lymphoma, older patients, population‐based, survival, treatment

## Abstract

Treatment for patients > 60 years with classical Hodgkin lymphoma (cHL) is problematic; there is no gold standard, and outcome is poor. Using the Swedish Lymphoma Registry, we analysed all Swedish patients diagnosed with cHL between 2000 and 2014 (*N* = 2345; median age 42 years; 691 patients were >60 years). The median follow‐up time was 6.7 years. Treatment for elderly patients consisted mainly of ABVD or CHOP, and the younger patients were treated with ABVD or BEACOPP (with no survival difference). In multivariable analysis of patients > 60 years, ABVD correlated with better survival than CHOP (*p* = 0.027), and ABVD became more common over time among patients aged 61–70 years (*p* = 0.0206). Coinciding with the implementation of FDG‐PET/CT, the fraction of advanced‐stage disease increased in later calendar periods, also in the older patient group. Survival has improved in cHL patients > 60 years (*p* = 0.027), for whom ABVD seems superior to CHOP.

## 1 INTRODUCTION

Younger patients with classical Hodgkin lymphoma (cHL) generally have a favourable prognosis, and in patients ≤ 60 years, recent debates on therapy for advanced‐stage cHL have largely revolved around ABVD (doxorubicin, bleomycin, vinblastine, dacarbazine) versus BEACOPP‐esc (bleomycin, etoposide, doxorubicin, cyclophosphamide, vincristine, procarbazine and prednisone).[1, 2, 3, 4]

In the older patient, the prognosis is much poorer,[5, 6, 7, 8] men have a worse prognosis than women in all ages [9], and a gold standard for treatment of patients > 60 years is yet to be established.

The CHOP (cyclophosphamide, doxorubicin, vincristine and prednisone) regimen has, during 2000–2017, been used to treat elderly cHL patients > 70 years in Sweden. In a small Norwegian retrospective single centre study, the CHOP regimen showed promising overall survival (OS) and progression‐free survival at 3 years of 79% and 76%, respectively.[10] ABVD is a standard treatment for younger patients and has also been tried in older patients. When used to treat elderly patients ABVD has a higher incidence of toxicity, in particular bleomycin‐related pulmonary toxicity.[11] More intensive chemotherapy combinations like BEACOPP have been associated with unacceptable toxicity and are generally not recommended for patients > 60 years.[12] With the introduction of brentuximab vedotin and immune checkpoint inhibitors in relapsed patients, the option of moving those treatments into first‐line has emerged. The B‐CAP (brentuximab vedotin, cyclophosphamide, doxorubicine and prednisone) regimen is such an attempt.[13]

Older patients and patients with severe comorbidities and social problems are often excluded from clinical trials.[14] Previous population‐based studies of adult cHL patients have been performed in Sweden [15, 16, 17] and elsewhere.[18, 19, 20] In the study performed by Bjorkholm, patients diagnosed between the years 1973 and 2014 demonstrated improved relative survival (RS) over time in all patient subgroups, including the elderly.[17] However, the outcome for the older patient is still dismal, especially in men, with limited difference in trends over time, and it was demonstrated that greater attention to the development of therapies for the older cHL patients is needed. Population‐based studies provide a true, real‐world, picture of treatment outcome and survival but continuous evaluations are needed, following changes in treatment standards, to identify unmet medical needs.

In the present study, we have analysed the difference in treatment outcome and patient characteristics with focus on the older patient group > 60 years.

## 2 METHODS

### 2.1 Swedish registries

Every clinician and pathologist/cytologist in Sweden is obliged by law to report each occurrence of cancer to the Swedish Cancer Registry (SCR).[21] The SCR includes diagnosis, gender, date of birth, date of diagnosis and the hospital where the diagnosis was made. The SCR and its diagnostic classification were introduced in 1958. By the 1990s, the Swedish Lymphoma Group commissioned the Swedish Lymphoma Registry (SLR), and it was launched nationwide on 1 January 2000.[22, 23] According to yearly reports, the coverage in SLR compared to the SCR has been 95%–97% since its initiation.[22] The SLR has been validated, showing a 95% agreement between the diagnosis reported to the SLR and the local patient files.[22] In June 2007, additional prognostic factors were added to the report form, allowing the calculation of the International Prognostic Score (IPS).[9] For residents in Sweden, date of death is centrally registered in the Causes of Death Registry, [24, 25] from which survival data were obtained.

**Key points**

- Outcome in classical Hodgkin lymphoma patients > 60 years of age is still poor.
- ABVD seems superior to CHOP in patients > 60 years.

### 2.2 Patients

All Swedish patients with newly diagnosed cHL and registered in the SLR from 1st January 2000 to 31st December 2014 were included in the analysis. Patients were not excluded due to comorbidities. All patients were observed from date of diagnosis until death, emigration or end of follow‐up (28th February 2015). Information on first‐line therapy, age, gender, WHO performance status, Ann Arbor stage, lactate dehydrogenase, B symptoms and IPS factors were obtained from the SLR. Sweden is divided into six Healthcare Regions, each with a population of 0.9–2.3 million people and a tertiary lymphoma centre, but cHL patients are treated also in smaller hospitals in every Region. In general, cHL patients are treated according to national guidelines, [26] which were first introduced in 1985 and since then regularly updated. This study was approved by the Ethics Committee, Stockholm.

### 2.3 An overview of treatment for HL in Sweden during 2000–2014

#### 2.3.1 Patients ≤ 60 years

Patients with stage I‐IIA and no risk factor (bulky disease, erythrocyte sedimentation rate > 50 mm or more than two involved sites) were given two courses of ABVD and in the beginning of the period followed by radiotherapy (RT) to 30 Gy or, in the latter part of the period, RT to 20 Gy in 2 Gy fractions, while those with any risk factor were treated with four ABVD followed by RT to 30 Gy or 29.75 Gy in 1.75 Gy fractions. Stage IB patients were treated with four ABVD plus RT to 30/29·75 Gy or as advanced‐stage disease (IIB‐IV). In the beginning of the studied period, RT was given as limited field RT (i.e., modified involved field) which gradually was replaced by involved site/node RT as radiological imaging techniques were refined. Patients with stage IIB‐IV disease, ≤60 years and IPS ≤ 2 were recommended 6–8 courses of ABVD [9] while those ≤ 60 and IPS > 2 were recommended six BEACOPPesc or eight BEACOPP‐14. In January 2011, when the RATHL trial [3] was launched in Sweden, all centres adopted its algorithm: six courses of ABVD with interim 18F‐fluourodeoxyglucose‐positron emission tomography/computer tomography (FDG‐PET/CT) after two courses, and those who did not show complete metabolic remission switched to BEACOPP‐14. This became the standard for patients with IPS ≤ 2 for the remainder of the study period (alongside six BEACOPPesc or eight BEACOPP‐14 for IPS > 2).

#### 2.3.2 Patients 61–70 years

Patients aged 61–70 with stage I‐IIA disease received treatment according to the same guidelines as patients ≤ 60 years. For stage IIB‐IV, patients aged 61–70 years were recommended six‐eight courses of ABVD.

#### 2.3.3 Patients ≥ 71 years

Stage I‐IIA without risk factors was treated with two cycles of CHOP followed by 30 Gy LF‐RT/INRT/ISRT in 2 Gy fractions, whereas stage I‐IIA with any risk factor received four cycles of CHOP and 30 Gy RT. Stage IB was either given four cycles of CHOP and 30 Gy RT or six cycles of CHOP. Advanced stages were recommended six cycles of CHOP as standard treatment; however, some centres also used ABVD in patients > 70.

### 2.4 Survival analysis

Survival of patients diagnosed with cHL was estimated using univariable Kaplan‐Meier and multivariable Cox OS analysis [27, 28] as well as univariable and multivariable RS analysis.[29, 30] From Swedish population life tables stratified by sex, age and calendar period, expected survival was assessed using the Ederer II method.[31] RS ratios (RSR) were estimated for patients in the three calendar periods and in four age groups (≤45, 46–60, 61–70 and ≥71 years). Multivariable models for excess mortality were calculated using the expectation‐maximization algorithm, employing a smoothed baseline excess hazard, yielding excess‐mortality rate‐ratios as estimates.[32] The excess‐mortality rate‐ratio denotes excess mortality (the difference between observed and expected mortality in the general population). The RS calculations were performed using R software (Maja Pohar Perme [2013]: relsurv, R package version 2.0‐4 [http://CRAN.R‐project.org/package = relsurv]); all other calculations with Stata version 9.2 (StataCorp, College Station, TX, USA).

## 3 RESULTS

A total of 2345 cHL patients (1257 men and 1088 women) were diagnosed in Sweden from year 2000 through 2014 (Table 1). Of all the patients, 294 were 61–70 years and 397 were ≥71 (Table 2). The median age at diagnosis was 42 years and increased over time (*p* = 0.010). The proportion of nodular sclerosis cases decreased (*p* = 0.003). There were no changes in WHO performance status or B symptoms over time. The median follow‐up time was 6.7 years (2000–2004, 12.8 years; 2005–2009, 7.5 years; 2010–2014, 2.8 years). Complete information on treatment was available in 1466 patients (417 patients ≥ 61 years). The IPS score, available from June 2007, predicted OS (*p* < 0.00005); however, because of its late adoption into the registry, IPS could not be used in multivariable analyses.

**TABLE 1:** Clinical characteristics – all patients

				Period						
		2000–2004 (*N* = 731)		2005–2009 (*N* = 800)		2010–2014 (*N* = 814)		Total (*N* = 2345)		
Characteristic		Number	%	Number	%	Number	%	Number	%	Univariable analysis with respect to overall survival HR (95% CI)
Age, years										
	Median (range)	38 (16–93)	43 (16–99)	45 (16–94)	42 (16–99)					
	16–45	408	55.8	420	53.1	414	51.0	1242	53.2	1
	46–60	126	17.2	131	16.6	144	17.7	401	17.2	2·89 (2.13–3.91
	61–70	78	10.7	108	13.7	108	13.3	294	12.6	7.80 (5.95–10.23)
	71–99	119	16.3	132	16.7	146	18.0	397	17.0	19.73 (15.53–25.07)
Histology subtype										
	NS	460	62.9	478	59.8	452	55.5	1390	59.3	1
	Not NS	167	22.8	203	25.4	187	23.0	557	23.8	2.09 (1.72–2.53)
	Unspecified	104	14.2	119	14.9	175	21.5	398	17.0	2.67 (2.18–3.29)
Sex										
	Female	355	48.6	362	45.3	371	45.6	1088	46.4	1
	Male	376	51.4	438	54.8	443	54.4	1257	53.6	1.36 (1.15–1.61)
Ann Arbor stage										
	I	121	16.8	110	14.0	98	12.3	329	14.3	1
	II	326	45.3	332	42.4	338	42.6	996	43.4	0.59 (0.44–0.78)
	III	159	22.1	182	23.2	157	19.8	498	21.7	1.62 (1.23–2.12)
	IV	113	15.7	159	20.3	201	25.3	473	20.6	1.97 (1.50–2.60)
B symptoms										
	Absent	384	54.3	408	52.0	432	53.7	1224	53.3	1
	Present	323	45.7	377	48.0	372	46.3	1072	46.7	2.10 (1.77–2.49)
Advanced stage disease (IIB, III, and IV)		393	55.0	467	60.0	474	59.7	1334	58.3	2.24 (1.85–2.71)
										
WHO performance										
	0	454	62.1	529	66.1	528	64.9	1511	64.4	1
	1	189	25.9	179	22.4	203	24.9	571	24.3	3.65 (3.00–4.44)
	≥ 2	88	12.0	92	11.5	83	10.2	263	11.2	9.24 (7.49–11.39)
Other IPS factors										
	Age over 45 years	323	44.2	371	46.9	398	49.0	1092	46.8	8.26 (6.60–10.33)
	Hb < 10·5 g/dl	–	–	53	17.3	133	17.3	186	17.3	3.24 (2.42–4.35)
	Albumin <40 g/l	–	–	193	65.4	491	66.6	685	66.3	3.46 (2.29–5.25)
	WBC > 15/nl	–	–	32	10.5	67	8.7	99	9.2	1.26 (0.81–1.96)
	B‐cells < 8% or < 0·6/nl	–	–	40	14.3	87	12.1	127	12.7	3.02 (2.15–4.25)
IPS										
	0 to 2	–	–	169	52.8	425	56.9	594	53.9	1
	3 to 7	–	–	151	47.2	322	43.1	508	46.1	6.36 (4.53–8.91)

**TABLE 2:** Clinical characteristics – patients > 60 years

Characteristics	ABVD		CHOP		Other		None		Total	
Age, years	No.	%	No.	%	No.	%	No.	%	No.	%
61–70	112	58	51	26	26	13	4	2	294	43
71–99	9	4	145	65	40	18	30	13	397	57
Sex										
Female	42	24	91	52	24	14	17	10	304	44
Male	79	33	105	43	42	17	17	7	387	56
Histology, subtype										
NS	51	33	70	45	24	16	9	6	257	37
Non‐NS	43	28	78	50	24	15	11	7	258	37
Unspecified	27	25	48	45	18	17	14	13	176	25
Ann Arbor stage										
I	29	48	36	36	3	5	11	11	121	17
II	37	34	51	51	11	10	6	5	173	25
III	26	23	52	52	25	22	4	4	188	27
IV	26	24	47	47	23	21	9	8	167	24
Missing	3	14	32	32	4	18	8	36	44	6
B‐symtoms										
No	64	38	76	45	18	11	12	7	316	46
Yes	57	24	117	49	46	19	17	7	353	51
Missing	0	0	3	30	2	20	5	50	22	3
WHO performance										
0	78	45	70	40	20	12	5	3	278	40
1	35	23	87	58	20	13	7	5	238	34
>2	7	8	37	41	25	28	21	23	161	23
Missing	1	20	2	40	1	20	1	20	14	2
IPS										
0‐2	27	41	28	42	8	12	3	5	105	28
3–7	47	25	90	49	28	15	20	11	264	72
Radiation therapy										
Yes	54	26	95	45	29	14	33	16	223	32
No	67	33	101	49	37	18	1	0	215	31
Missing	na	na	na	na	na	na	na	na	253	37

### 3.1 Survival analysis

OS at 3 and 5 years was 83% (95% confidence interval [CI], 81%–84%) and 79% (95% CI, 77%–81%), respectively. OS at 3 years according to age, stage and sex is shown in Table 3. Survival did not change over time, neither in limited (*p* = 0.67) nor advanced‐stage (*p* = 0.76) disease (Figure S1B). In patients 61–70 years, 3‐ and 5‐year OS was 71% and 64% and in patients ≥ 71 years, 42% and 33%.

**TABLE 3:** Overall survival at 3 years

	Overall survival, at 3 years			
Category All patients	2000–2009	95% CI	2010–2014	95% CI
≤45	97%	95%–98%	96%	94%–98%
46–60	89%	85%–92%	89%	81%–93%
61–70	68%	61%–74%	79%	69%–86%
≥71	40%	34%–46%	47%	37%–56%
Limited stages				
≤45	98%	96%–99%	98%	94%–99%
46–60	97%	92%–99%	91%	77%–97%
61–70	87%	77%–93%	96%	76%–99%
≥71	63%	51%–72%	71%	53%–83%
Advanced stages				
≤45	96%	94%–97%	95%	90%–97%
46–60	83%	76%–88%	87%	75%–93%
61–70	57%	47%–66%	73%	60%–82%
≥71	34%	26%–41%	44%	32%–55%
Advanced stages, men				
≤45	94%	90%–97%	91%	83%–95%
46–60	83%	73%–89%	87%	72%–94%
61–70	59%	46%–69%	67%	51%–79%
≥71	39%	29%–49%	36%	21%–51%
Advanced stages, women				
≤45	98%	94%–99%	99%	93%–100%
46–60	83%	69%–91%	87%	65%–96%
61–70	55%	39%–68%	82%	61%–92%
≥71	25%	15%–37%	54%	35%–69%

**FIGURE 1:**
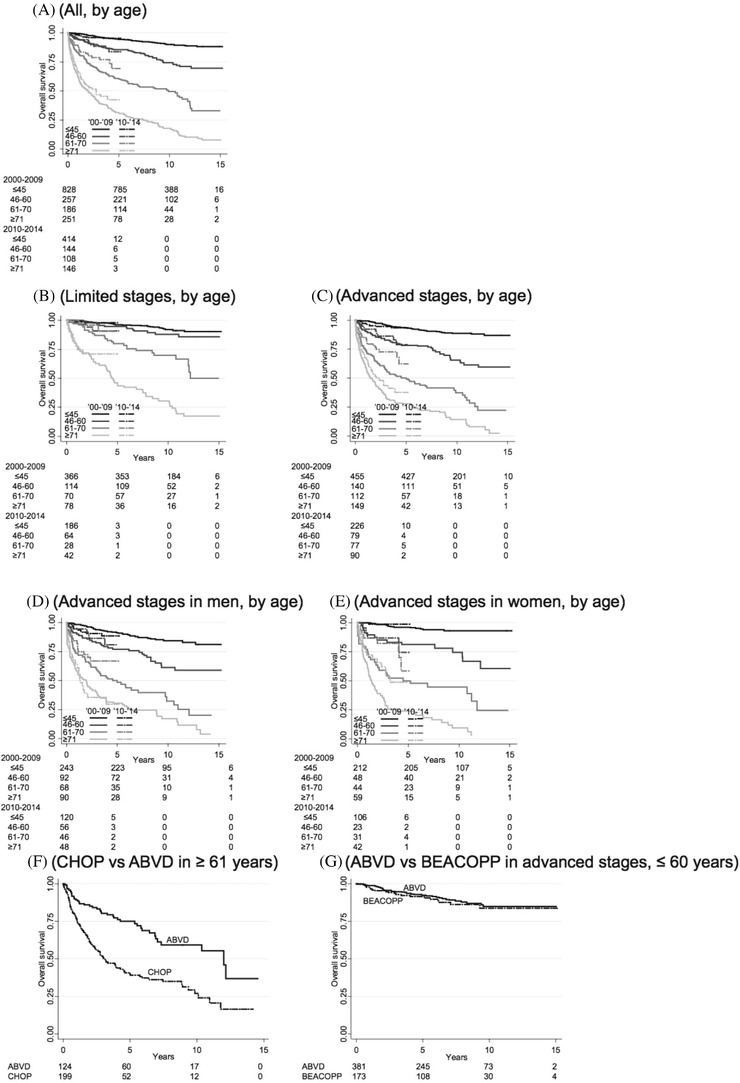
Overall survival. By (A) calendar period and age for all patients, (B) calendar period and age in limited (I‐IIA) stages, (C) calendar period and age in advanced (IIB‐IV) stages, (D) calendar period and age in men with advanced stages, (E) calendar period in women with advanced stages, (F) CHOP or ABVD in patients ≥ 61 years of age, (G) BEACOPP or ABVD in patients ≤ 60 years with advanced stages

In patients ≥ 61 years, OS was better in those diagnosed between 2010 and 2014 than in those diagnosed between 2000 and 2009 (*p* = 0.027); this was not seen in patients ≤ 60 years (*p* = 0.49; Figure 1A). The improvement in patients ≥ 61 was due to better OS in advanced‐stage disease (*p* = 0.011; Figures 1B‐1C), and in women (*p* = 0.005; Figures 1D and 1E, Table 4), but not in patients with limited‐stage disease (*p* = 0.34). There were no survival differences between the Healthcare Regions.

**TABLE 4:** Multivariable models

		Overall survival		Relative survival	
Subset	Factor	HR	95% CI	RR	95% CI
Women > 60 years stage IIB‐IV	WHO > 1	3.34	2.23–4.99	4.26	2.63–6.89
(*N* = 174)	Age > 70 years	2.11	1.37–3.25	1.61	0.97–2.65
	Period 2010–2014	0.53	0.33–0.86	0.49	0.28–0.87
Patients > 60 years	WHO > 1	2.88	1.94–4.27	3.31	2.10–5.23
(*N* = 304)	Age > 70 years	1.40	0.95–2.07	1.16	0.71–1.90
	Stage IIB‐IV	2.37	1.52–3.70	3.39	1.57–7.28
	CHOP vs. ABVD	1.80	1.14–2.85	2.30	1.24–4.28

Competing, non‐significant factors: Histology, LDH, B symptoms (and, in the latter analysis, male sex).

Abbreviation: CI, confidence interval; HR, hazard ratio; RR, relative risk.

The women's IPS scores did not change over time, and 26% were high‐risk (IPS > 2) both 2000–2009 and 2010–2014. Furthermore, there was no change in their performance status, histology or the incidence of B symptoms (data not shown). The fraction of stage IV cases increased in the last calendar period (from 18% to 25%; *p* < 0.00005); this was seen over all categories: female (from 15% to 22%; *p* = 0.003), male (from 21% to 28%; *p* = 0.006), ≤60 years old (from 16% to 22%; *p* = 0.001) and >60 years old patients (from 23% to 31%; *p* = 0.044).

### 3.2 Chemotherapy regimens

Chemotherapy regimens by age, sex and calendar periods are presented in Table 5. In multivariable analysis of patients > 60 years, CHOP compared to ABVD was an independent adverse factor for OS (*p* = 0.039; Table 4, see also Figure 1F). When repeating the multivariable analysis only in patients aged 61–70 years, CHOP was still inferior to ABVD (HR, 1.72; *p* = 0.039). Only nine patients ≥ 71 years had received ABVD, why statistical significance could not be achieved (*p* = 0.097).

**TABLE 5:** Chemotherapy regimens according to subsets

		Age group				
Subset All patients	Therapy	≤45	46–60	61–70	≥71	All ages
	None	0%	0%	2%	13%	2%
	Reduced	3%	2%	9%	17%	6%
	CHOP	0%	2%	26%	65%	14%
	ABVD	80%	77%	58%	4%	65%
	BEACOPP	17%	18%	5%	1%	13%
Limited stages	None	0%	1%	2%	18%	2%
	Reduced	3%	1%	8%	7%	3%
	CHOP	0%	1%	14%	67%	8%
	ABVD	95%	97%	76%	7%	85%
	BEACOPP	2%	0%	0%	0%	2%
Advanced stages	None	0%	0%	2%	8%	2%
	Reduced	3%	4%	8%	21%	7%
	CHOP	0%	2%	33%	67%	17%
	ABVD	68%	61%	50%	3%	52%
	BEACOPP	29%	33%	7%	1%	22%
Limited stages	None	0%	0%	0%	9%	1%
2000–2009	Reduced	4%	5%	9%	22%	8%
	ABVD	68%	60%	41%	3%	52%
	CHOP	0%	3%	43%	65%	17%
	BEACOPP	29%	32%	7%	1%	22%
Advanced stages	None	0%	0%	4%	7%	2%
2010–2014	Reduced	1%	2%	6%	20%	6%
	CHOP	0%	0%	18%	70%	18%
	ABVD	69%	64%	64%	2%	53%
	BEACOPP	30%	34%	8%	2%	21%
Advanced stages	None	0%	0%	0%	9%	2%
2000–2009	Reduced	5%	7%	12%	20%	9%
Men	CHOP	0%	3%	38%	69%	18%
	ABVD	62%	55%	42%	2%	47%
	BEACOPP	34%	34%	8%	0%	25%
Advanced stages	None	0%	0%	3%	3%	1%
2010–2014	Reduced	1%	0%	7%	30%	7%
Men	CHOP	0%	0%	13%	60%	13%
	ABVD	65%	67%	67%	3%	55%
	BEACOPP	33%	33%	10%	3%	24%
Advanced stages	None	0%	0%	0%	8%	1%
2000–2009	Reduced	2%	0%	4%	24%	6%
Women	CHOP	0%	3%	54%	59%	16%
	ABVD	75%	69%	38%	5%	58%
	BEACOPP	23%	28%	4%	3%	19%
Advanced stages	None	0%	0%	5%	10%	3%
Women	Reduced	0%	6%	5%	10%	4%
2010–2014	CHOP	0%	0%	25%	80%	23%
	ABVD	73%	59%	60%	0%	52%
	BEACOPP	27%	35%	5%	0%	18%

Patients ≤ 60 years with advanced‐stage disease treated with BEACOPP and ABVD showed identical OS in univariable (Figure 1G) and multivariable analysis (adjusted for the same covariates as in Table 3; *p* = 0.51). In patients with complete information and IPS‐score > 2, there was a trend (*p* = 0.33) for better OS with BEACOPP. The patients treated with BEACOPP were older than those treated with ABVD (median age, 33 vs. 30 years; *p* = 0.012).

### 3.3 RS

**FIGURE 2:**
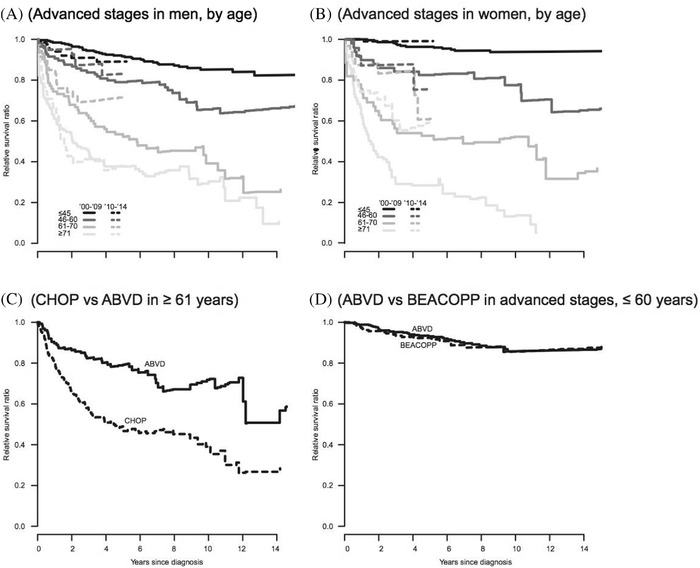
Relative survival. By (A) calendar period and age in men with advanced stages, (B) calendar period in women with advanced stages, (B) CHOP or ABVD in patients ≥ 61 years of age, (C) BEACOPP or ABVD in patients ≤ 60 years with advanced stages

RS was stable between the calendar periods (Figures 2 and S2), except for an improvement in elderly women with advanced stage disease diagnosed between 2010 and 2014 (Figure 2B; Table 1). CHOP seems inferior to ABVD in the older patient group, while BEACOPP was equal to ABVD in younger patients with advanced‐stage disease (Figure 2), also after adjustment for clinical factors (Table 4).

## 4 DISCUSSION

Using the SLR, we have performed a retrospective analysis of all 2345 patients registered with cHL in Sweden from 2000 through 2014, with focus on patients > 60 years. ABVD seems superior to CHOP for older patients, a finding that should be interpreted with caution even if it was significant in multivariable analysis due to the obvious risk of selection bias. Agreeing with previous reports,[10, 11, 15, 33] prognosis remains poor for patients > 60 years. This is due to their inability to tolerate BEACOPP [12] or ABVD,[11] higher co‐morbidity [7] and differences in lymphoma biology (more mixed cellularity, Epstein‐Barr virus positivity and infradiaphragmatic disease).[7] FDG‐PET/CT was gradually introduced, and a stage migration was seen over time as increased number of patients presenting with stage IV disease in the last calendar period, also in the older patient group.

In Sweden, between 2000 and 2014 there was no improvement in survival except in patients > 60 years. Improved survival, especially in elderly women, was seen in nationwide Swedish studies of patients diagnosed with follicular lymphoma (FL) and advanced nodular lymphocyte predominant HL [34, 35] during the same time period.

In Sweden, CHOP has been used for patients over 70 years of age since 2000, based on the results from a Norwegian study.[10] For those aged 60–70 years, ABVD was recommended. In our material, CHOP (compared with ABVD) was an independent adverse factor for OS in patients ≥ 61 years, when adjusted for available risk factors. Hence, ABVD is preferable for those fit to receive such treatment. Given that ABVD provides better outcome than CHOP and that bleomycin toxicity is a major problem in older patients receiving more than two cycles of ABVD,[11, 36] the option of excluding bleomycin is tempting. There are studies indicating that the omission of bleomycin has a relatively small impact on efficacy.[3, 37] AVD (doxorubicin, vinblastine, dacarbazine) can also be given with G‐CSF (granulocyte colony‐stimulating factor) support without the risk of lung injury. A study in which bleomycin was removed or reduced in 53 of 147 patients > 60 years, mainly due to lung toxicity, came to the same conclusion,[38] and in the Swedish national guidelines [26] AVD (doxorubicin, vincristine and dacarbazine) is recommended to patients > 70 years as first‐line treatment since 2017. In Sweden AVD is also used to treat patients 61–70 years with co‐morbidity, not fit for ABVD.

Other alternatives for treating the elderly are being explored, for example, in a co‐operative phase II study between the German Hodgkin study group and the Nordic lymphoma group, where B‐CAP (brentuximab vedotin, cyclophosphamide, doxorubicin and prednisone) was used for those fit to receive combination chemotherapy. Patients not fit for combination chemotherapy were treated with brentuximab vedotin monotherapy.[13] Unfortunately, preliminary results have not reached a sufficient level to form a new standard.[39] This may in part be due to the CHOP backbone that brentuximab vedotin was combined with.

Younger patients with IPS 0–2 were treated with 6–8 ABVD, and those with IPS 3–7 received either 6–8 ABVD or BEACOPP‐14/esc. In recent years, the ABVD treatment has been PET‐guided, with escalation to BEACOPP‐14/esc for PET‐positive patients.

Currently, the German standard for advanced stage disease is 4–6 [4] courses of BEACOPPesc [2], and in multiple studies, survival has been superior for the BEACOPP‐regimens.[2, 4, 40, 41, 42, 43, 44, 45]

In a previous Swedish study of long‐term survival after cHL, where data from patients treated between 1992 and 2009 (*N* = 1947, age 18–59 years) were analysed, the survival of relapse‐free patients was similar to that of the general population, while the relapsed patients had an excess mortality 19 times higher compared to the relapse‐free.[16]

In our material, patients ≤ 60 years with advanced‐stage disease treated initially with BEACOPP‐14/esc versus ABVD showed identical OS in univariable and multivariable analysis. These data indicate that a PET‐guided ABVD approach is suitable for a proportion of the advanced‐stage patients. However, the IPS score, available from June 2007, predicted OS (*p* < 0.00005), suggesting that a proportion of those with high‐risk score might need a more aggressive approach from the start. Since BEACOPP was only given to high‐risk patients, according to IPS, it could be argued that the identical OS might suggest that BEACOPP is beneficial for this group. For the patients > 60 years, no PET‐guided approach is available due to the lack of any evidence‐based escalation‐regimen.

The strengths of our study are that it is population‐based, consecutive and based on real‐life data. No regional differences were seen in treatment or survival. One weakness is the bias in the selection of chemotherapy regimens used, especially for CHOP versus ABVD in older patients but also in ABVD versus BEACOPP‐14/esc for the younger. Some patients starting with ABVD escalated to BEACOPP‐14/esc due to positive PET/CT. Another weakness is the limited follow‐up time for patients in the latest calendar period, reducing the possibility to distinguish toxicity form anti‐lymphoma effect. Furthermore, the SLR lacks information on relapse, and cause of death; however, the RSR analysis abrogates that. Because the IPS was introduced only recently into the registry, a proper comparison between ABVD and BEACOPP‐14/esc when indicated (≤60 years, advanced‐stage and IPS > 2) could not be satisfactorily performed.

In conclusion, ABVD seems superior to CHOP for patients > 60 years who can tolerate this regimen and since the study period, the treatment of patients > 70 years and older frail patients, has been changed from CHOP to AVD in clinical routine in the Nordic countries. Outcome was not improved over time from 2000 to 2014, except in older patients, but further investigation and longer follow‐up are needed to confirm this observation. The implementation of FDG‐PET/CT coincided with a stage migration seen in real life as an increase in stage IV disease, also in the older patient group (Table 1).

## CONFLICT OF INTEREST

Daniel Molin has received honoraria from Roche, Merck, Bristol‐Myers Squibb and Takeda. Björn Engelbrekt Wahlin has received research funding from Roche and Gilead, and he has been a consultant for Roche and Gilead. Lotta Hansson has received research grant support from Gilead and Janssen‐Cilag and honoraria from Abbvie. Ingrid Glimelius has received honoraria from Janssen‐Cilag. Marzia Palma has received research grant support from Beigene and Takeda. None of the other authors have any relevant conflict of interest to disclose.

## AUTHOR CONTRIBUTIONS

Björn Engelbrekt Wahlin wrote the manuscript and interpreted the data together with Ninja Övergaard and Daniel Molin and performed the statistics together with Stefan Peterson. Stefan Peterson made statistical analyses, especially relative survival and participated in the writing of the manuscript. Evangelos Digkas and Ingrid Glimelius participated in the writing of the manuscript. Ingemar Lagerlöf, Ann‐Sofie Johansson, Marzia Palma, Lotta Hansson, Johan Linderoth and Christina Goldkuhl contributed to the Swedish Lymphoma Registry and participated in the writing of the manuscript.

## Supporting information



Supporting Information

## Data Availability

The data that support the findings of this study are available on request from the corresponding author. The data are not publicly available due to privacy or ethical restrictions.
