# Supplementary material for: Real‐world data on treatment concepts in classical Hodgkin lymphoma in Sweden 2000–2014, focusing on patients aged >60 years
Source: EJHaem. 2021 May 6;2(3):400–12. doi: 10.1002/jha2.202 (PMC9175745; doi:10.1002/jha2.202)
Supplement: Supplementary file 1 — Supporting Information [file JHA2-2-400-s001.docx]

# Supplement

**Table I Relative survival at 3 years.**

| Category | Relative survival, at 3 years | | | |
| --- | --- | --- | --- | --- |
|  | 2000-2009 | | 2010-2014 | |
| All patients |  | 95% CI |  | 95% CI |
| ≤45 | 97% | 96%-98% | 96% | 94%-98% |
| 46-60 | 90% | 86%-94% | 90% | 84%-96% |
| 61-70 | 70% | 64%-78% | 81% | 73%-90% |
| ≥71 | 46% | 40%-54% | 54% | 44%-66% |
| Limited stages |  |  |  |  |
| ≤45 | 98% | 96%-99% | 98% | 96%-100% |
| 46-60 | 98% | 96%-101% | 92% | 84%-101% |
| 61-70 | 90% | 82%-99% | 100% | 92%-107% |
| ≥71 | 72% | 61%-86% | 82% | 67%-102% |
| Advanced stages |  |  |  |  |
| ≤45 | 96% | 94%-98% | 95% | 92%-98% |
| 46-60 | 84% | 78%-90% | 87% | 79%-96% |
| 61-70 | 59% | 51%-70% | 75% | 65%-87% |
| ≥71 | 39% | 31%-48% | 50% | 38%-65% |
| Advanced stages, men |  |  |  |  |
| ≤45 | 94% | 92%-97% | 91% | 86%-97% |
| 46-60 | 84% | 76%-92% | 88% | 78%-99% |
| 61-70 | 61% | 50%-75% | 70% | 56%-86% |
| ≥71 | 45% | 35%-58% | 41% | 27%-63% |
| Advanced stages, women |  |  |  |  |
| ≤45 | 98% | 96%-100% | 99% | 97%-101% |
| 46-60 | 84% | 74%-95% | 88% | 75%-102% |
| 61-70 | 56% | 43%-73% | 84% | 70%-100% |
| ≥71 | 29% | 19%-45% | 60% | 44%-83% |

**Supplementary figure legends.**

**Supplementary figure 1. Overall survival in Sweden 2000-2014.**

By (A) calendar period all patients, (B) calendar period and limited (I-IIA) and advanced (IIB-IV) stages, (C) calendar period in men, (D) calendar period in women.

**Supplementary figure 2. Relative survival in Sweden 2000-2014.**

By (A) calendar period all patients, (B) calendar period and limited (I-IIA) and advanced (IIB-IV) stages, (C) calendar period in men, (D) calendar period in women.

**Supplementary figure 1.**


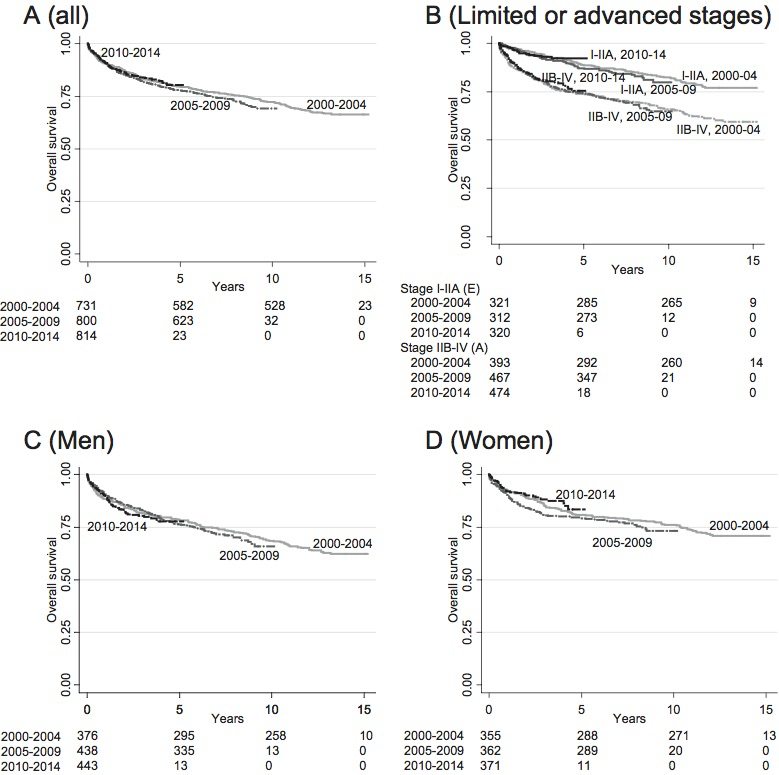


**Supplementary figure 2.**

A (all) B (Limited or advanced stages)

0

2

4

6

8

10

12

14

0.0

0.2

0.4

0.6

0.8

1.0

o

i

t

a

r

l

a

v

i

v

r

u

s

e

v

i

t

a

l

e

R

2010−2014

2005−2009

2000−2004

0

2

4

6

8

10

12

14

0.0

0.2

0.4

0.6

0.8

1.0

o

i

t

a

r

l

a

v

i

v

r

u

s

e

v

i

t

a

l

e

R

I-IIA, 2010−14

IIB-IV, 2010−14

I-II

A, 2005−09

IIB-IV, 2005−09

I-IIA, 2000−04

IIB-I

V, 2000−04

Years since diagnosis Years since diagnosis

0

2

4

6

8

10

12

14

0.0

0.2

0.4

0.6

0.8

1.0

C (Men)

Years since diagnosis

o

i

t

a

r

l

a

v

i

v

r

u

s

e

v

i

t

a

l

e

R

2010−2014

2005−2009

2000−2004

0

2

4

6

8

10

12

14

0.0

0.2

0.4

0.6

0.8

1.0

D (Women)

Years since diagnosis

o

i

t

a

r

l

a

v

i

v

r

u

s

e

v

i

t

a

l

e

R

2010−2014

2005−2009

2000−2004
